# A Trail to Diagnosis—Finding the Primary Lesions of Bone Metastasis

**DOI:** 10.7759/cureus.23814

**Published:** 2022-04-04

**Authors:** Ejaz Shah, Waqas Azhar, Saliha Saleem

**Affiliations:** 1 Internal Medicine, Saint John Hospital, Springfield, USA; 2 Hematology/Oncology, Southern Illinois University School of Medicine, Springfield, USA

**Keywords:** hypercalcemia, unusual presentation, bone metastases, esophageal cancer, hip pain

## Abstract

This case reports an interesting case of hip pain. A 70-year-old male came to the hospital with lethargy and right hip pain. X-ray of the right hip was concerning for impending pathological fracture of right femur. Blood work was significant for hypercalcemia. He was managed with fluids, bisphosphates, and right hip arthroplasty. A bone biopsy was taken. His initial workup included an X-ray skeletal survey and computer tomography (CT) of the chest and abdomen to diagnose etiology of the right hip lesion. An X-ray skeletal survey showed multiple osteolytic bone lesions very suspicious for multiple myeloma. CT chest and abdomen did not show any concerning relevant findings. However, bone biopsy resulted as poorly differentiated adenocarcinoma of pancreatic or gastrointestinal origin. Magnetic resonance imaging (MRI) of the abdomen/pancreatic protocol was done, which showed normal pancreas and associated ducts. Later he underwent endoscopy showing stricture at the lower esophagus, whose biopsy confirmed the diagnosis of poorly differentiated adenocarcinoma with esophagus as primary site. Further staging workup was completed by positron emission tomography (PET) scan. It was stage four at the time of diagnosis. Right hip pain was secondary to bone metastasis from esophageal cancer (EC). The primary lesion was not noticeable on CT imaging despite the evident extensive metastasis, challenging the diagnosis. He was offered palliative radiation therapy for bone metastasis and associated pain. Unfortunately, he continued to have recurrent hospital admissions with other medical conditions, and his physical health declined rapidly. He died within a few months after diagnosis.

## Introduction

Esophageal cancer (EC) is the eighth most common cancer worldwide. EC is considered the sixth most common cause of cancer death [1]. 30% of patients are diagnosed as metastatic at the time of diagnosis and therefore associated with a poor prognosis [2]. The five-year survival rate of people with EC diagnosis is, on average, 20% [3]. Survival is primarily determined by the stage at the time of diagnosis. It is approximately 41% with stage one or localized tumor and decreased to 5% with stage four or metastatic disease [4].

There are two main histological types of EC, adenocarcinoma and squamous cell carcinoma (SCC). SCC is the most prevalent histological type worldwide [5].

However, the incidence of adenocarcinoma has increased significantly and is currently the leading cause of EC in the United States, accounting for almost 80% of the cases [6]. EC is asymptomatic at earlier stages. In advanced stages, it can cause progressive dysphagia (for solids first and then liquids), refractory gastroesophageal reflux disease despite proton pump inhibitors, and unintentional weight loss [4]. It has four stages based on tumor size, nodal involvement, and metastasis. Treatment largely depends on the stage. Stage four has limited treatment options, including palliative radiotherapy and chemotherapy. Here, we present a case of EC with an uncommon presentation of right hip pain. His tumor size was not significant; however, it was metastatic at the time of diagnosis, making his cancer stage four.

## Case presentation

A 70-year-old Caucasian male with a medical history of hypothyroidism, hypertension, thoracic aneurysm, and left tonsillar SCC was admitted to the hospital with right hip pain. He was also noted to be lethargic on examination but with no focal neurological deficit. He had a prior history of smoking. His tonsillar cancer was treated more than 10 years back, and he was considered cured then.

**Figure 1:**
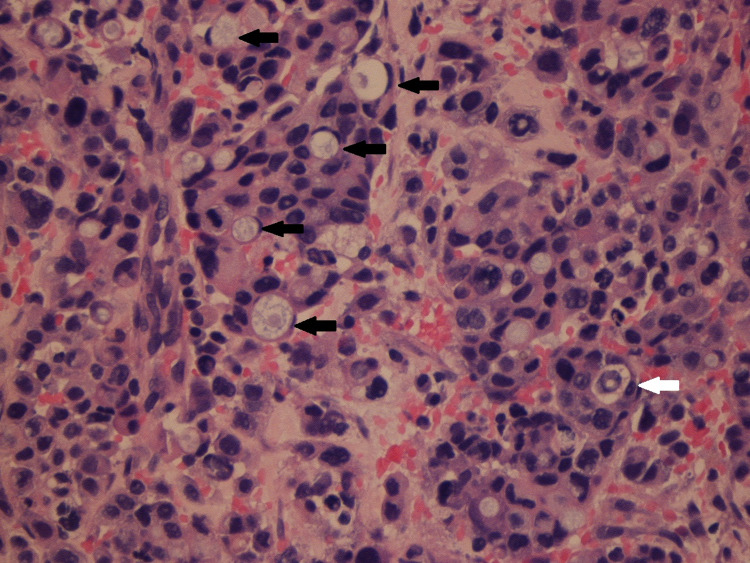
Poorly differentiated adenocarcinoma with signet cells replacing normal bone marrow in the sacrum (shown with arrows).

**Figure 2:**
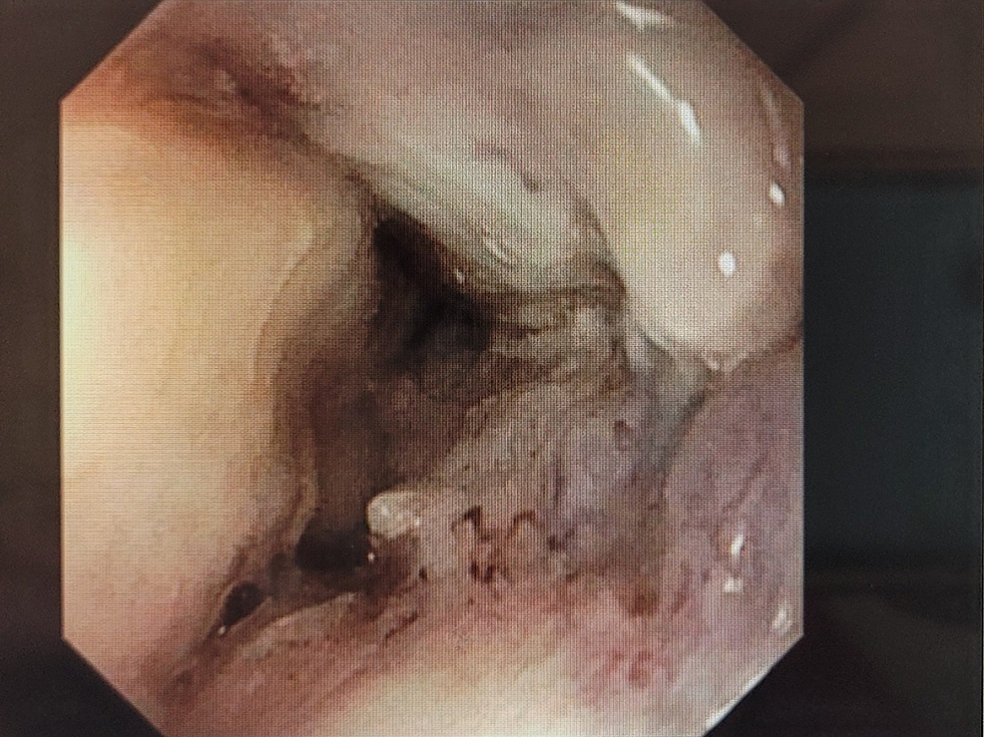
Stenosis at lower esophagus found in endoscopy.

**Figure 3:**
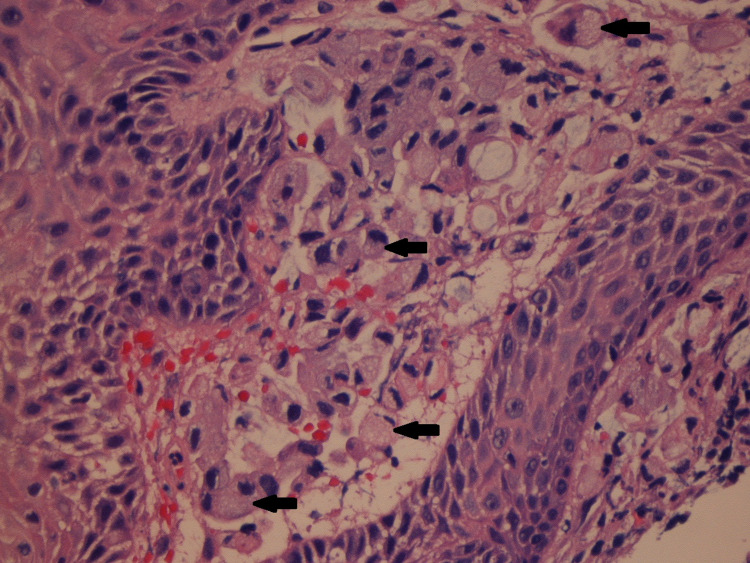
Signet cells infiltrating through lamina propria in the esophagus (marked with arrows).

**Figure 4:**
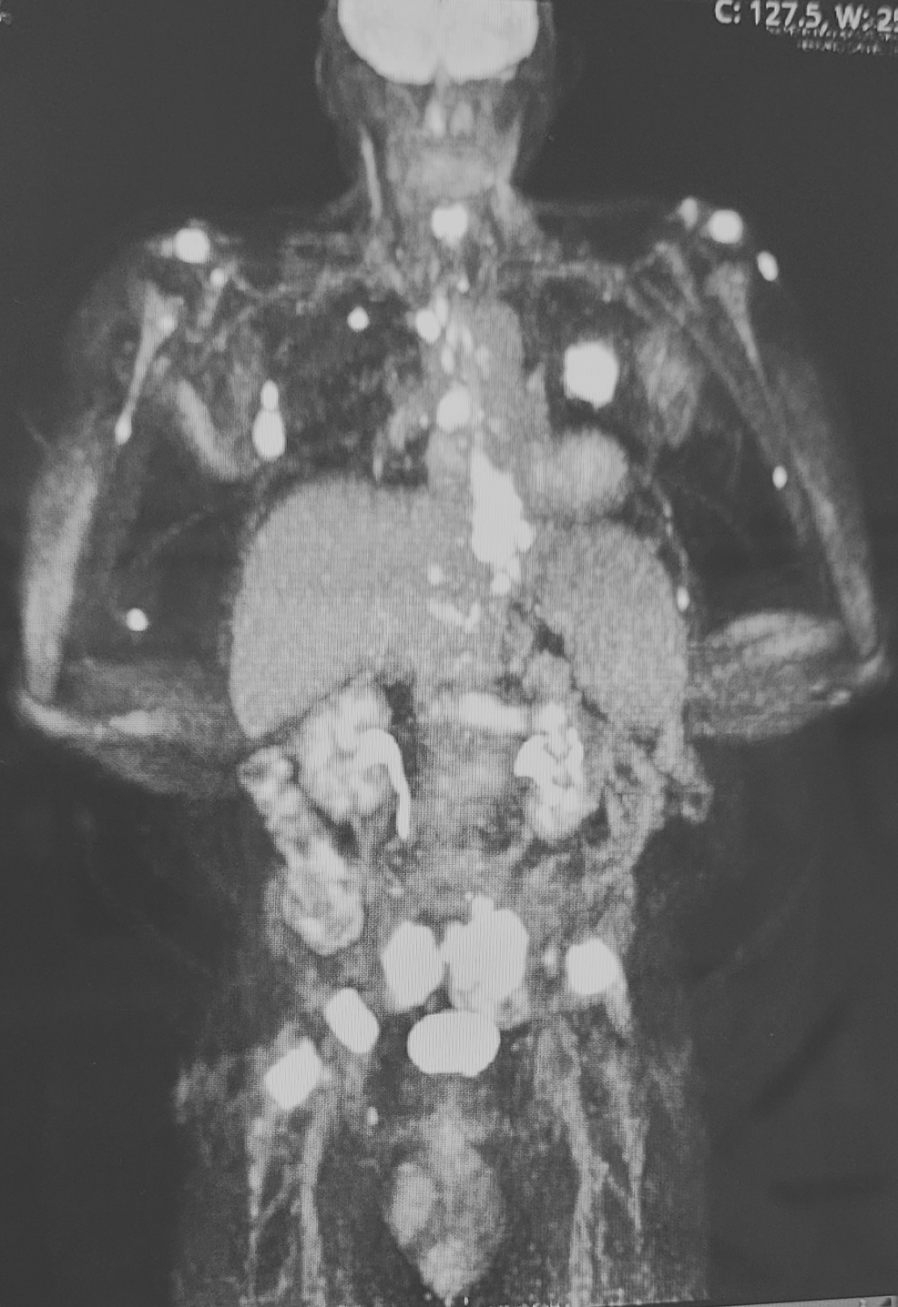
PET scan showing distant metastases above and below the diaphragm. PET: positron emission tomography

Initial workup in the hospital included basic blood work and imaging. Blood work was concerning for acute kidney injury, creatinine 1.44 (normal range: 0.7-1.3 mg/dl), hypercalcemia with total calcium of 12.8 (normal range: 8.4 -10.5 mg/dl), and ionized calcium 1.7 (normal range: 1.15-1.33 mmol/l). He was started on intravenous fluid and was given intravenous bisphosphonates and calcitonin. His renal functions and calcium levels improved in subsequent days. Clinically his lethargy improved, and he was back to his baseline mental status as calcium levels normalized. While his workup for hypercalcemia and right hip pain was under process, his right hip X-ray showed an impending fracture of the right femur. MRI of the right hip showed a large right femoral head and neck junction lesion, which was at high risk for a pathological fracture. He underwent urgent right hip arthroplasty, and a bone biopsy was also done. A skeletal survey was ordered, which showed multiple lytic lesions in ribs, lumbar spine, sacrum, pelvis, right femoral neck, left iliac crest, and right scapula; findings were concerning for multiple myeloma versus metastatic disease. Given the prior history of tonsillar cancer, CT soft tissue neck was done, which showed normal postsurgical changes in the head and neck, with no evidence of residual or recurrent malignancy. He then underwent CT-guided bone marrow biopsy from the left iliac crest and sacrum. Both intraoperative bone biopsy and bone marrow biopsy results were concerning for poorly differentiated adenocarcinoma (Figure 1). Immunohistochemical stains revealed that the malignant cells were positive for cytokeratin AE1/AE3, Ber-EP4, CA19.9, CAM5.2, CEA, EMA, and MOC-31 with partial positive staining for CK20 and weak positive staining for CDX2. The malignant cells were negative for CK7 and NKX3.1. The findings supported the diagnosis of poorly differentiated adenocarcinoma. The findings were not specific for the site of primary tumor origin but suggested pancreaticobiliary origin. Other gastrointestinal primary sites, including stomach and intestinal primary, were other possibilities. Given his immunostains, he underwent an MRI abdomen-pancreatic protocol where no distinct pancreatic mass lesion was identified. CT chest and abdomen did not show any obvious mass lesion. He underwent endoscopy, which revealed a malignant appearing, intrinsic stenosis (Figure 2). Biopsies were taken, demonstrating infiltrating poorly differentiated adenocarcinoma with esophagus as the primary site of origin (Figure 3). He underwent a positron emission tomography (PET) scan of the body to complete a staging workup, showing multiple sites of abnormally increased 18F-fluorodeoxyglucose (FDG) accumulation involving both soft tissue and bone occurring both above and below the diaphragm, consistent with widespread malignancy (Figure 4).

He was started on palliative radiation therapy while other stains were ordered. However, progressively his oral intake declined, and he was started on percutaneous endoscopic gastrostomy (PEG) tube feedings. He was then discharged to rehabilitation. He presented again in two weeks with shortness of breath and was diagnosed with pulmonary embolism, pneumonia, and pleural effusions. He received his second session of radiation. Despite being on anticoagulation, diuretics, and antibiotics, respiratory status continued to decline. Goals of care were discussed with the patient and family, and a decision was made to continue with comfort measures only. He expired in the following week with acute respiratory failure.

## Discussion

EC is known for its extensive metastasis and poor survival rates. SCC is a more prevalent histological type in developing countries. Risk factors for SCC mainly include age (peak 70s), black race, achalasia, tobacco use, and alcohol consumption. In the United States and other developed countries, adenocarcinoma is increasing in incidence. Risk factors for adenocarcinoma include male sex, white race, gastroesophageal reflux disease (GERD), Barrett metaplasia, and obesity. The incidence has increased by eightfold since 1975, and it is believed to be secondary to the increased prevalence of GERD, Barrett esophagus, and obesity [4,7].

EC has four stages according to the tumor, nodes, and metastasized (TNM) staging. Staging accounts for T tumor depth, N nodal involvement, and M metastasis. Tumor depth is further elaborated as T1 to T4 depending on the involvement of different layers of the esophagus. This is referred to as carcinoma in situ. N is mentioned as N0 or N1 based on the absence or presence of nodal involvement, respectively. M is mentioned as M0 and M1 based on the absence and presence of metastasis, respectively. EC is well known to be highly metastatic, given its multiple modes of metastases. It can spread through local invasion, lymphatic spread, and hematogenous. Lack of serosa helps with the local spread of EC to adjacent structures, extensive lymphatic drainage of the esophagus helps spread to thoracic regions, and the hematogenous route helps spread to distant organs like the liver and bones [2,8-9]. Common metastatic sites for EC include lymph nodes, liver, lung, and bone. Investigations utilized to stage EC include EUS, CT of the chest and abdomen, and PET scan. EUS is sensitive for the T stage, CT chest and abdomen especially helps identify the thoracic spread of EC, and PET scan helps identify distant metastases. Tumor markers for EC are an area of advancing research. Tumor markers might help in early diagnosis and assist in assessing tumor response to therapy [10,11]. Prognostic factors that are considered to be related to EC include platelet count, d-dimer, nutrition status, and tumor length [12-14].

Treatment options are based on staging and include endoscopic mucosal resection, esophagectomy, radiation, and chemotherapy, used as a single modality or combination. Despite advancements in treatment options, the prognosis remains poor [4,7,11]. There are no current recommendations to help screen or prevent EC. Surveillance endoscopy is recommended for diagnosed Barrett’s esophagus; however, it is based on expert opinion only [15].

This case report exemplifies another case of metastatic esophageal carcinoma, where the patient presented without the common presenting symptoms of dysphagia, acid reflux, or weight loss. Interestingly his CT chest and abdomen did not show any obvious lesion or mass, but it was already extensively metastatic and categorized as stage four at the time of diagnosis. All the necessary workup was done for him during the hospital stay in a span of a few weeks, including endoscopic ultrasonography (EUS), CT chest and abdomen, PET scan, and biopsy of both bone and esophageal lesion. However, the excessive disease burden and poor physical health made him decline rapidly. Toward the last few weeks, he developed shortness of breath and was diagnosed with submassive pulmonary embolism, community-acquired pneumonia, and diastolic heart failure with pleural effusions and vascular congestion. He received two sessions of palliative radiotherapy for his bone pain. Being aware of his poor prognosis and progressive deteriorating health, he and his family decided to be comfort care. In the next few days, he expired of acute respiratory failure.

In the literature review to date, there is one case report of esophageal adenocarcinoma presenting with extensive bone metastases. It presented as osteolytic and osteoblastic lesions in the femur, humerus, ribs, cervical, and lumbar vertebrae [16]. Other similar yet limited case reports are for esophageal SCC presenting as solitary bone metastases. Sites mentioned are femur, tibia, iliac, cervical vertebra, rib, and calcaneus [17-18].

## Conclusions

Bone metastases are common in stage four cancer. However, finding the primary lesion could be an interesting task. This man had an unusual presentation of EC. His primary lesion was not evident on the CT scan, despite the disease being already widespread.
